# UV Light-Curable Epoxy Coatings with Natural Plant-Based Fillers—Evaluation of Antibacterial and Functional Properties

**DOI:** 10.3390/ma18235464

**Published:** 2025-12-04

**Authors:** Wojciech Żyłka, Barbara Pilch-Pitera, Katarzyna Krawczyk, Ewa Ciszkowicz, Beata Grabowska, Artur Bobrowski

**Affiliations:** 1 Faculty of Exact and Technical Sciences, Institute of Materials Engineering, University of Rzeszow, Pigonia 1, 35-310 Rzeszow, Poland; 2 Department of Polymers and Biopolymers, Faculty of Chemistry, Rzeszów University of Technology, al. Powstańców Warszawy 12, 35-029 Rzeszów, Poland; 3 Pigments and Coatings, Fraunhofer Institute for Manufacturing Engineering and Automation IPA, Allmandring 37, 70569 Stuttgart, Germany; katarzyna.krawczyk@ipa.fraunhofer.de; 4 Department of Biotechnology and Bioinformatics, Faculty of Chemistry, Rzeszów University of Technology, al. Powstańców Warszawy 12, 35-029 Rzeszów, Poland; ewa.ciszkowicz@prz.edu.pl; 5 Department of Foundry Process Engineering, Faculty of Foundry Engineering, AGH University of Krakow, al. A. Mickiewicza 30, 30-059 Kraków, Poland; beata.grabowska@agh.edu.pl (B.G.); arturb@agh.edu.pl (A.B.)

**Keywords:** epoxy coatings, plant-based fillers, antibacterial properties, surface potential SKP

## Abstract

This article presents the results of research on UV-curable epoxy coatings developed with selected plant modifiers such as garlic (*Allium sativum*), turmeric (*Curcuma longa*), common nettle (*Urtica dioica*), and privet (*Ligustrum vulgare*). This study aimed to evaluate the influence of these natural components on the functional properties of UV-cured coatings and to assess their potential as bio-based modifiers. The coatings were formulated using Epidian^®^ 5 epoxy resin, a safe and non-toxic material approved for food-contact applications, and cured with a commercial cationic photoinitiator. Their mechanical, surface, optical, and antibacterial properties were investigated. The results showed that all plant-based additives modified both the mechanical and esthetic characteristics of the coatings; however, garlic demonstrated outstanding antibacterial activity, achieving nearly complete inhibition of *Staphylococcus aureus* growth with a reduction rate of 99.998%. These findings highlight that natural modifiers, especially garlic, can serve as highly effective functional components, while future work should focus on implementing these coatings for surfaces exposed to bacteria, such as public utility items and shop, hospital, sports, and rehabilitation equipment.

## 1. Introduction

In recent years, the concept of sustainable development and the principles of green chemistry have become one of the main directions of progress in materials engineering and polymer chemistry. Among coating technologies, UV-curable systems have attracted particular attention due to their rapid cross-linking at ambient temperature, low energy consumption, and the absence of volatile organic compound (VOC) emissions. These advantages make them a more sustainable alternative to conventional solvent-based or thermally cured coatings [1,2,3]. Despite their numerous benefits, most commercial UV-curable coatings are still based on epoxy or acrylate resins of petrochemical origin, which raises concerns about their long-term impact on the environment and human health [4,5]. In response to these challenges, new paint and varnish formulations are being developed that utilize renewable raw materials, including plant-derived compounds, as modifiers or active components of polymer matrices. The incorporation of natural additives does not completely eliminate the environmental impact of the synthetic matrix but represents a step toward more sustainable solutions by reducing the amount of synthetic components and toxic additives or biocides [5,6,7]. Plant-derived materials have attracted increasing attention as renewable and functional modifiers of polymer systems. Plant extracts and powders obtained from garlic (*Allium sativum*), turmeric (*Curcuma longa*), nettle (*Urtica dioica*), and privet (*Ligustrum vulgare*) contain a wide spectrum of bioactive compounds such as flavonoids, polyphenols, alkaloids, and essential oils [8,9,10,11,12,13,14,15,16]. These substances can influence the chemical and physical properties of polymer coatings in various ways. For instance, phenols and flavonoids enhance oxidative stability and UV resistance of the cured network; essential oils may modify surface energy and improve hydrophobicity; whereas sulfur compounds from garlic and curcumin from turmeric exhibit natural antibacterial activity. At the same time, their presence may affect the mechanical properties or color of coatings, which justifies the need for a detailed assessment of the influence of individual fillers on the final material performance [11,13,17,18]. It should be emphasized that plant fillers are not homogeneous materials—their composition and properties depend on the plant species, harvesting time and location, as well as drying and grinding conditions. They may also exhibit limited resistance to light and elevated temperature, as well as a specific color or odor, which can be a significant limitation in coating applications, particularly where high surface esthetics are required. Four plant species—garlic, turmeric, nettle, and privet—were selected for this study due to their local availability and diverse chemical composition, which allows for the evaluation of the effects of different groups of bioactive compounds on coating properties. Garlic (*Allium sativum*) is a source of sulfur compounds (mainly allicin) with strong antibacterial activity [14]; turmeric (*Curcuma longa*) contains curcumin, a pigment with well-known antioxidant and photoprotective properties [11]; nettle (*Urtica dioica*) is rich in flavonoids and polyphenols that may improve UV resistance [12,13]; while privet (*Ligustrum vulgare*) contains iridoids and saponins, whose influence on polymer systems has not yet been studied [7,12]. The selection of chemically diverse plants enabled a comprehensive evaluation of how natural substances of different chemical structures can modify the physicochemical and functional properties of epoxy coatings. It should also be noted that although some of these raw materials (e.g., privet) may exhibit biological toxicity when ingested, their components are permanently immobilized in the polymer matrix in coating applications, eliminating any risk of user contact. The development of durable and biocompatible coatings is of particular importance for applications involving frequent human contact, such as rehabilitation equipment, medical devices, or astronaut training systems, where surface hygiene and resistance to microbial growth are crucial [19,20,21]. Therefore, the aim of this study was to investigate the effect of selected plant-based fillers (garlic, turmeric, nettle, and privet) on the physicochemical and antibacterial properties of UV-curable epoxy coatings. The study evaluated whether these natural additives can impart bioactive functionality to coatings without compromising their mechanical and esthetic properties, thereby contributing to the development of safer and more sustainable coating materials.

## 2. Materials and Methods

Dried leaves of garlic (*Allium sativum*, GAR, Rzeszow, Poland), turmeric (*Curcuma longa*, TUR, Kania, India), common nettle (*Urtica dioica*, NET, Rzeszow, Poland), and privet (*Ligustrum vulgare*, PRI, Rzeszow, Poland) were used in this study.

Nettle and privet leaves were harvested from a local farm in June 2025 and dried. Garlic (bulbs) and turmeric (rhizomes) were purchased locally in dried form. The garlic was locally grown, and the turmeric came from India.

The raw materials were finely ground and subsequently sieved through a 100 µm sieve to obtain a uniform particle size fraction for paint formulation. The selected, ground plant-based modifiers were thoroughly mixed with Epidian^®^ 5 epoxy resin (Sarzyna Chemicals, Nowa Sarzyna, Poland) in the following proportions: 95 g of resin, 5 g of plant modifier, and 0.61 g of a photoinitiator—a mixture of triarylsulfonium hexafluoroantimonate salts (50% solution in propylene carbonate, Aldrich, Buchs, Switzerland). The qualitative and quantitative composition of the coatings is presented in Table 1. The mixture was applied to Q-panels R-36 substrates using a standard applicator bar (gap thickness: 120 µm, Research and Production Laboratory of Rzeszow University of Technology, Rzeszow, Poland), ensuring the formation of a uniform coating layer for further testing. Subsequently, the coatings were cured using a Dymax UVC-5 Compact Light-Curing Conveyor System equipped with a mercury lamp (Dymax, Torrington, CT, USA).

Plant fillers were exposed to an accelerated weathering test in a Xenon Test Chamber (Xentest 2200) by TestAn (Anticorr, Gdańsk, Poland). Samples were tested according to the following parameters: irradiance at 340 nm 0.51 W/m^2^, black pattern temperature (BPT) 30.0 °C, relative humidity 15.0% RH (dry phase), daylight filters. All samples were tested for 24 h. Color change measurements were taken before and after the chamber test.

A Thermobalance TGA/SDTA 851e (Mettler-Toledo, Greifensee, Switzerland) was used for thermogravimetric analysis (TGA) of plant fillers. The analysis parameters were as follows: temperature range from 25 to 600 °C, heating rate of 10 °C/min, nitrogen atmosphere, gas flow 50 cm^3^/min, sample weight ~5 mg, 150 μL open alumina pans. The FT-IR spectra of the cured coatings were recorded using a Thermo Scientific Nicolet 6700 FT-IR spectrophotometer (Thermo Fisher Scientific, Waltham, MA, USA) equipped with a helium–neon (HeNe) laser in the range of 600–4000 cm^−1^ with a resolution of 4 cm^−1^. The data are presented as transmittance (%) versus wavenumber (cm^−1^). Surface roughness of the cured coatings was measured using a Mar Surf PSI profilometer (Mahr, Göttingen, Germany) in accordance with PN-EN ISO 12085 [22]. Roughness profile measurements were taken at three different locations on each sample. Scratch resistance was tested using a manual Clemen scratch tester (Elcometer, Nieuwegein, The Netherlands) in accordance with PN-EN ISO 1518-1 [23]. Adhesion to the substrate was evaluated using the cross-cut test according to PN-EN ISO 2409 [24]. The coating was incised with a multi-blade cutter (BYK-Gardner, Geretsried, Germany) equipped with six blades spaced 3 mm apart, oriented perpendicular to the surface. Adhesive tape was then applied over the incisions, left in place for five minutes, and subsequently removed. Adhesion quality was evaluated visually and rated on a six-point scale, where 0 indicates excellent adhesion (smooth incision edges with no coating loss) and 5 indicates poor adhesion (surface damage exceeding 65% of the grid area). Relative hardness was measured using a König pendulum hardness tester (BYK-Gardner, Geretsried, Germany) in accordance with PN-EN ISO 1522 [25]. This parameter is expressed as the number of pendulum oscillations before coming to rest; the higher the number, the harder the surface. A reference glass plate (175 oscillations, BYK-Gardner, Geretsried, Germany) was used as a benchmark representing the maximum attainable surface hardness. Gloss was evaluated using a micro-TRI-gloss µ tester (BYK-Gardner, Geretsried, Germany) in accordance with PN-EN ISO 2813 [26]. Film thickness was determined using the same device according to PN-EN ISO 2808 [27]. Color parameters were determined using a Test-An 45/0 spectrophotometer (Anticorr, Gdańsk, Poland) in accordance with PN-ISO 7724 [28]. Measurements were performed under directional illumination at 45° ± 5°, using a CIE D65 standard illuminant in SCE (Specular Component Excluded) mode with a spectral interval of 10 nm. Color evaluation was based on the CIELAB color space parameters (L*, a*, b*).

where

- L*—lightness (0 = black, 100 = white);
- a*—green-red axis (− = green, + = red);
- b*—blue-yellow axis (− = blue, + = yellow).

The color difference (ΔE) was calculated according to Equation (1):

(1) $$\Delta E^{*}=\sqrt{\;{\Delta L}^{*2}{+\Delta a}^{*2}+{\Delta b}^{*2}\;}$$

The water contact angle was determined using the sessile drop method with an optical goniometer (Data Physics, model OCA 15, Filderstadt, Germany) in accordance with EN 828 [29]. Distilled water was used as the test liquid. Measurements were performed on samples with a surface roughness (Ra) close to 0.5 µm to minimize the influence of surface topography on the contact angle. A 1 µL droplet of the liquid was applied, and the contact angle was analyzed using SCA20U software Version 2. The surface free energy (SFE) was determined according to the Owens–Wendt method, based on the measured contact angles and the known surface tension values of the test liquids [30]. The antibacterial activity of the coating surfaces was evaluated in accordance with ISO 22196 [31] against Escherichia coli and Staphylococcus aureus. A 1 mL bacterial suspension (1–5 × 10^8^ CFU/mL) was mixed with 100 mL of agar solution at 45 ± 2 °C. The coated samples were placed in Petri dishes and moistened with sterile saline using cotton swabs (TZMO S.A., Toruń, Poland). Subsequently, 0.5 mL of the agar–bacteria mixture was applied onto each coating, forming a pseudo-biofilm layer not exceeding 1 mm in thickness. After solidification, the samples were incubated at 37 °C in a high-humidity incubator for 24 h to prevent drying. After incubation, both test and control samples were transferred to sterile beakers using flame-sterilized tweezers (Aldrich, Buchs, Switzerland). To each beaker, 4.5 mL of neutralizing broth was added (dilution 1:10). The beakers were sealed with Parafilm, sonicated, and vortexed for 1 min. Serial tenfold dilutions were then prepared by transferring 0.5 mL of solution from each step (A to B, B to C, and C to D) into 4.5 mL of neutralizing broth, yielding a final dilution of 1:10,000. For bacterial counting, each Petri dish containing solid agar was divided into three sections, and 25 µL of each diluted solution was spread using sterile spreaders (Aldrich, Buchs, Switzerland). After 24 h of incubation at 37 °C, the colony-forming units (CFUs) were counted. The antibacterial effect was calculated based on the difference in CFU counts between the reference coating (without antimicrobial agent) and the tested coating (with antimicrobial agent). Scanning Kelvin Probe (SKP) microscopy (Anfatec, Oelsnitz, Germany) was used to measure the potential distribution on the surface of the cured coatings.

## 3. Results

### 3.1. Fillers Characterization

**Figure 1:**
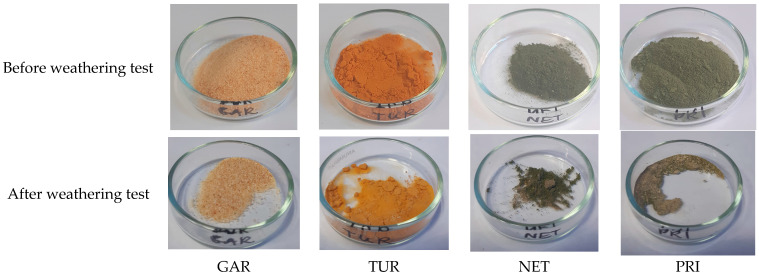
Plant-based fillers: garlic (*Allium sativum*, GAR), turmeric (*Curcuma longa*, TUR), common nettle (*Urtica dioica*, NET), and privet (*Ligustrum vulgare*, PRI) before and after the weathering test.

Plants fillers that demonstrate antibacterial activity in other applications were selected for this study to determine their effectiveness in epoxy coatings. Plant fillers have the advantage of being renewable, but their properties may vary depending on the harvest period, location, and growing conditions. Furthermore, they may have disadvantages such as poor light resistance or susceptibility to elevated temperatures. Their color and odor can also be problematic in coating applications. The dark color of the filler can interfere with dyeing coatings in light colors. The garlic used was light, creamy, turmeric was yellow, and nettle and privet were green (Figure 1).

#### 3.1.1. Light Stability

To assess light resistance, the samples were tested in an aging chamber with a xenon lamp, the radiation of which is closest to visible light. The appearance of the filler samples before and after the weathering test is shown in Figure 1, while the CIELAB color parameters and total color difference (ΔE) are presented in Table 2, Table 3, Table 4 and Table 5.

After the weathering test, a change in the CIELAB color parameters was observed for all the plant-based fillers. The samples exhibited a lighter color, which is reflected by a decrease in the L* value (a shift toward a whiter shade). The a* parameter increased in the case of garlic, indicating a shift toward a redder color. For turmeric, a decrease in the a* value occurred, indicating a shift toward green, while the increase in a* for nettle and privet indicates a reduction in the intensity of the green color. For GAR, TUR, and NET, the b* parameter decreased, meaning a reduction in the intensity of the yellow shade, whereas the increase in b* for PRI indicates a reduction in the intensity of the blue shade. The total color change (ΔE) was the smallest for garlic, which is almost imperceptible to the naked eye, while the remaining fillers exhibited a noticeable change toward lighter colors. Such a change may be advantageous when the paint needs to be colored in light shades.

#### 3.1.2. Thermal Stability

**Figure 2:**
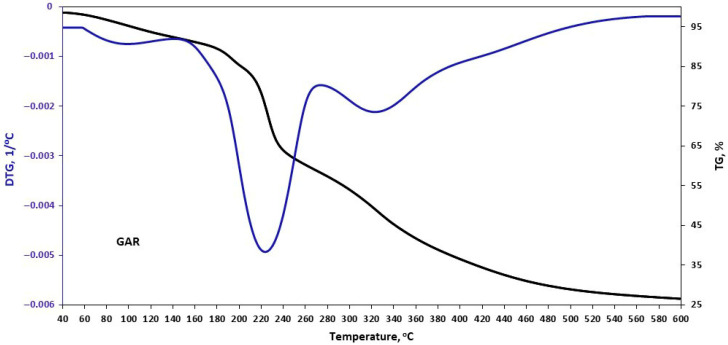
TGA curves of garlic (GAR).

**Figure 3:**
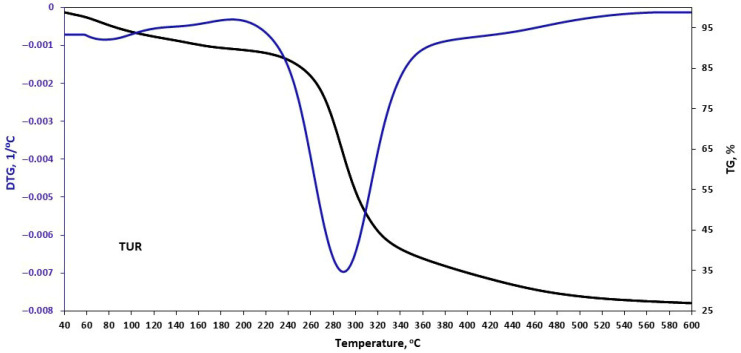
TGA curves of turmeric (TUR).

**Figure 4:**
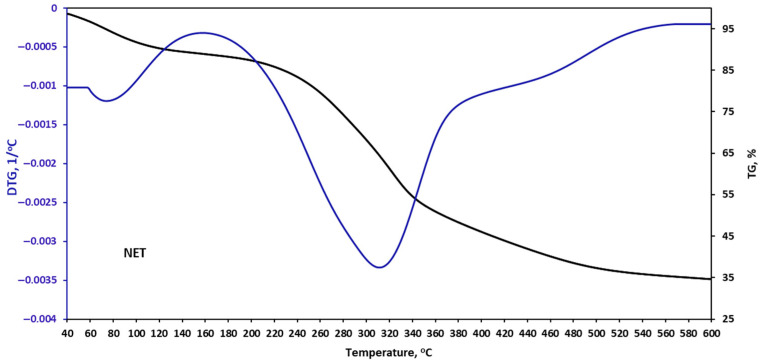
TGA curves of nettle (NET).

**Figure 5:**
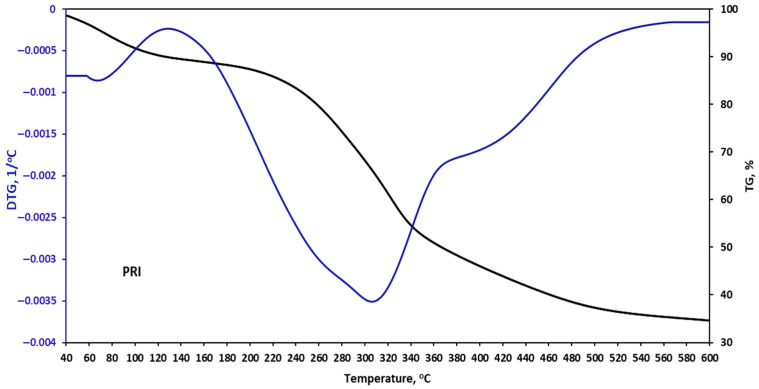
TGA curves of privet (PRI).

The thermal stability of the plant filler samples was assessed during controlled heating using a thermobalance. The obtained results are presented in Figure 2, Figure 3, Figure 4 and Figure 5 and in Table 6.

The initial stage of thermal decomposition of the plant fillers involves the evaporation of moisture. The water content of the tested samples was 6–8%. The thermogram of garlic powder showed that it was stable up to 150 °C. The onset temperature of garlic degradation was 150 °C, with a weight loss of 36% at the onset temperature. This is due to the degradation of lignin between 150 and 300 °C, caused by the cleavage of α- and β-aryl-alkyl-ether linkages that occur in this temperature range [32]. Allicin decomposition also takes place at this stage [33]. At temperatures above 300 °C, hemicellulose, cellulose, and other organic components decompose [34].

In the case of turmeric, the next mass-loss stage occurs at 190 °C and corresponds to lignin degradation. In this final stage, hemicellulose and cellulose also degrade [35].

Nettle powder degradation begins at 165 °C and corresponds first to lignin decomposition, followed by hemicellulose. Cellulose degradation also begins at this stage and is completed at approximately 480 °C. The mass loss at this stage is 57.2%, which is slightly higher than the 50.50% reported in the literature for nettle fibers [34]. This may be due to the heterogeneous composition of plants collected from different locations.

Privet degradation begins at 140 °C, indicating that it contains more lignin than garlic, turmeric, and nettle. The maximum degradation occurs at 310 °C, similar to the other tested fillers [36].

For PRI, a lower char residue is observed (24.8%), while TUR exhibits a 15% weight loss, which may be related to a higher content of thermally stable mineral components. It is worth noting that the thermal stability of plant-based fillers refers to the maximum temperature at which they resist decomposition. However, due to the heterogeneous composition of plants resulting from growing conditions, differences in the decomposition process may occur. All plant-based fillers tested are susceptible to degradation at temperatures above 140 °C, which can compromise the structural integrity of the coatings. Thermal stability is therefore a key factor when modifying polymer coatings. To prevent thermal degradation during production and application, it is recommended that the processing and service temperature does not exceed 140 °C.

It is worth noting that the thermal stability of plant-based fillers refers to the maximum temperature at which they remain resistant to decomposition. However, due to the heterogeneous composition of plants resulting from growing conditions, differences in the decomposition process may occur. All the plant-based fillers tested are susceptible to degradation at temperatures above 150 °C, which may compromise the structural integrity of the coatings. Thermal stability is therefore a key factor when modifying polymer coatings. To prevent thermal degradation during production and service, it is recommended that the processing and operating temperature does not exceed 150 °C.

### 3.2. Coatings Characterization

**Figure 6:**
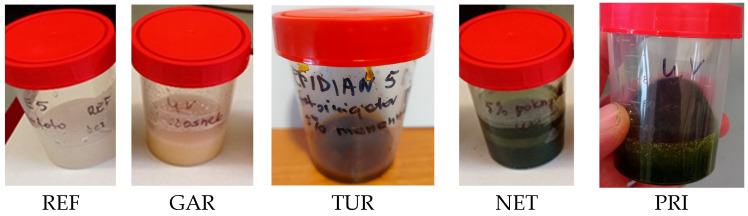
Prepared paint formulations: reference sample (REF) and modified with plant-based additives—garlic (*Allium sativum*, GAR), turmeric (*Curcuma longa*, TUR), common nettle (*Urtica dioica*, NET), and privet (*Ligustrum vulgare*, PRI).

The low-molecular-weight epoxy resin Epidian^®^ 5 was used to obtain a photocurable polymer coating. To initiate the photocuring reaction, a cationic photoinitiator—triarylsulfonium hexafluoroantimonate salt—was employed. This photoinitiator generates strong Brønsted acids under UV radiation, initiating the ring-opening cationic polymerization of epoxy groups. In contrast to radical photopolymerization, oxygen does not inhibit this process, and an inert atmosphere is not required. Moreover, cationic polymerization proceeds to greater depths and continues even after UV exposure—a phenomenon known as “dark polymerization” [37]. This is particularly advantageous when curing coatings on parts with complex geometries or greater thicknesses. The concentration range was limited to 5% by weight because preliminary screening studies suggested that higher concentrations increased viscosity and reduced coating integrity, while lower concentrations showed no measurable differences in antibacterial activity. Using a low-viscosity epoxy resin facilitated the dissolution of the photoinitiator and the dispersion of plant-based modifiers without the use of solvents. As a result, homogeneous liquid samples were obtained, as shown in Figure 6. These samples were then applied to test substrates, photocured under UV radiation, and subjected to further characterization.

#### 3.2.1. Chemical Structure Characterization of the Coatings

**Figure 7:**
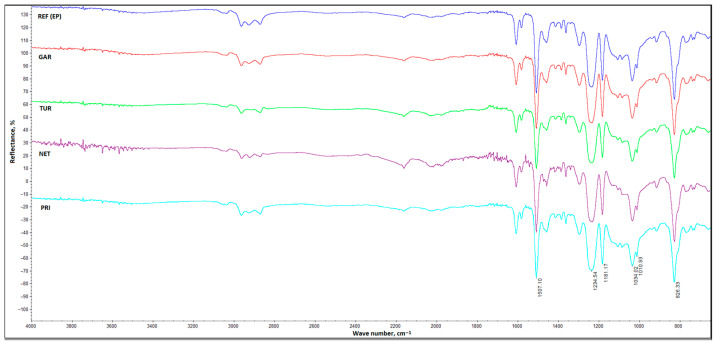
FT-IR spectra of the obtained coatings: REF, GAR, TUR, NET and PRI.

FT-IR analysis was used to characterize the chemical structure of the synthesized coatings. The FT-IR spectra of all investigated coatings are presented in Figure 7.

All spectra are nearly identical, showing mainly signals originating from the epoxy matrix. All FT-IR spectra display a broad peak at 3450 cm^−1^, which is assigned to the stretching vibration of OH groups. The peak at 3040 cm^−1^ is related to aromatic C–H stretching vibrations originating from the aromatic ring of 2,2-bis(p-hydroxyphenyl)propane, a typical epoxy resin raw material. Absorption bands in the range of 2800–3000 cm^−1^ indicate the presence of aliphatic groups (–CH_2_, –CH_3_), while signals at 1411 cm^−1^ are characteristic of the C–H vibrations of methyl groups. The three peaks at 1607, 1507, and 1462 cm^−1^ are related to C=C stretching vibrations of the aromatic ring. The peaks at 1234, 1181, and 1114 cm^−1^ are characteristic of aromatic C–H out-of-plane bending vibrations [38].

The characteristic C–O–C stretching vibrations are observed in the range of 1100–1200 cm^−1^. The weak peak at 910 cm^−1^ indicates the presence of a small amount of unreacted epoxy groups remaining after the cross-linking process. The intensity of this peak is comparable for all tested coatings, indicating that the plant fillers did not have a significant effect on the coating cross-linking process. The peak at 826 cm^−1^ is related to out-of-plane deformation vibrations of the 1,4-substituted aromatic ring originating from 2,2-bis(p-hydroxyphenyl)propane [39].

The signals derived from the plant fillers are not visible in the spectra of the coatings containing them, probably because they are obscured by the stronger signals originating from the epoxy matrix. Both the active compounds and the cell-wall components—cellulose, hemicellulose, and pectins—give signals in a similar spectral range because they contain groups such as OH, C–O–C, or CH_2_, which are also present in the epoxy matrix.

#### 3.2.2. Surface Roughness

**Figure 8:**
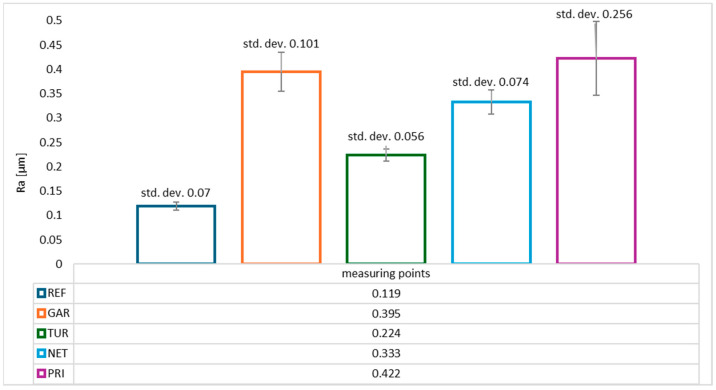
Average Ra surface roughness values (µm) of epoxy coatings modified with plant-based additives.

Figure 8 presents the surface roughness results expressed as the Ra parameter. The Ra values show a clear trend: the addition of natural modifiers increases surface roughness, which may influence functional properties such as gloss, interlayer adhesion, and resistance to soiling.

The lowest surface roughness was observed for the reference sample without the addition of plant-based modifiers (Ra ≈ 0.119 µm), indicating a uniform, smooth surface typical of unmodified epoxy resin. The highest average roughness was observed for coatings containing privet (Ra ≈ 0.422 µm) and garlic (Ra ≈ 0.395 µm). The elevated Ra values may result from irregular grinding or the presence of larger, insoluble particles in the plant powder. Modifiers derived from nettle and turmeric caused a moderate increase in surface roughness compared to the reference sample (Ra ≈ 0.333 µm and 0.224 µm, respectively). All Ra values remained below 0.5 µm, classifying the coatings as smooth surfaces.

**Figure 9:**
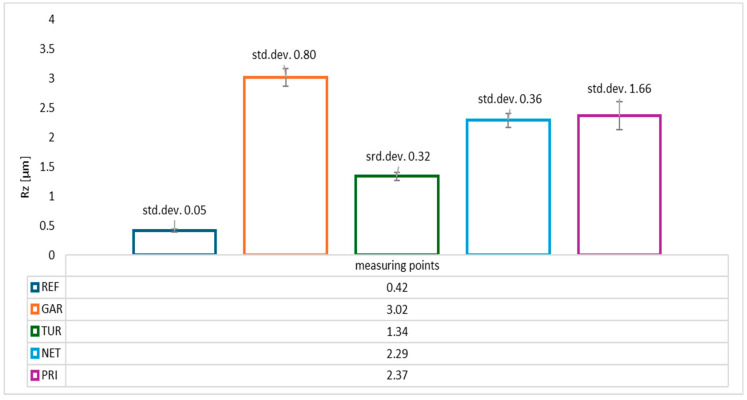
Average Rz surface roughness values (µm) of epoxy coatings modified with plant-based additives.

In addition to the average roughness (Ra), the maximum height of surface irregularities (Rz) was also measured, with the results presented in Figure 9.

The lowest Rz values were recorded for the reference sample without the addition of modifiers (Rz ≈ 0.42 µm), confirming a homogeneous, smooth surface typical of pure epoxy resin. The highest Rz values were observed for coatings containing garlic (Rz ≈ 3.01 µm) and the privet modifier (Rz ≈ 2.37 µm). The high values and large standard deviations, especially for privet, may indicate uneven distribution or the presence of larger solid particles within the coating. Coatings containing turmeric and nettle exhibited moderate Rz values (Rz ≈ 1.34–2.29 µm), with nettle showing noticeably higher variability among measurements. The highest variability was observed for the sample containing privet, suggesting surface structure irregularities—possibly resulting from particle agglomeration tendencies.

#### 3.2.3. Scratch Resistance

**Figure 10:**
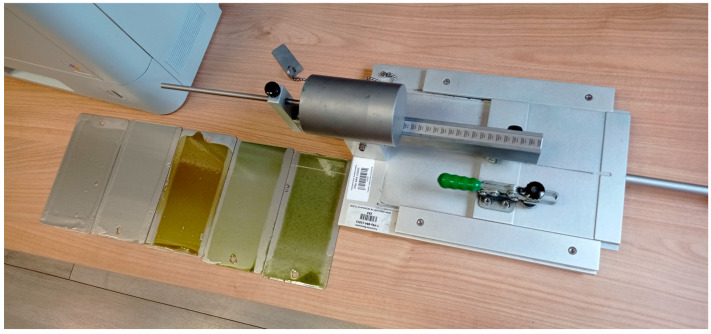
The test setup for the evaluation of scratch resistance in epoxy coatings using the Clemen method.

Figure 10 presents a photograph of plant-based epoxy coating samples during scratch resistance testing using a Clemen scratch tester equipped with a loading arm.

**Figure 11:**
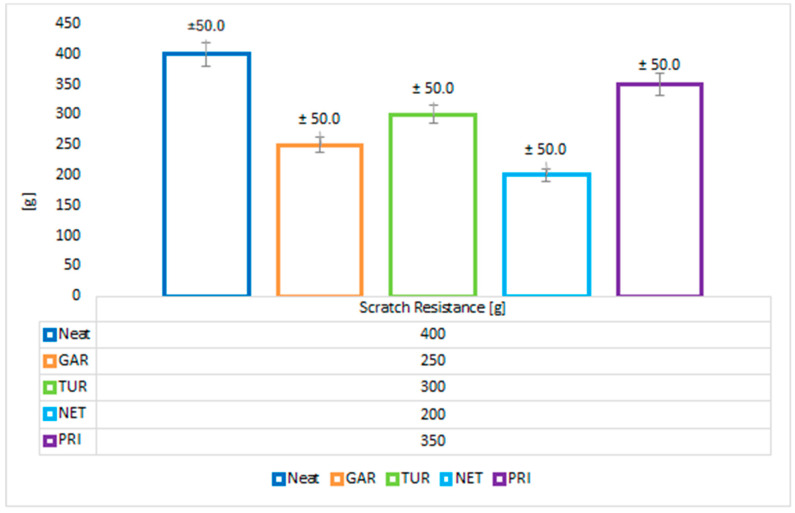
Scratch resistance of epoxy coatings modified with plant-based fillers.

The test results are presented in Figure 11. Scratch resistance testing revealed clear differences between the reference sample and coatings containing plant-based fillers. The highest resistance was recorded for the coating without the addition of plant-based modifiers (neat epoxy resin), reaching a value of 400 g, which indicates a compact, homogeneous, and well-crosslinked structure.

The addition of plant-based modifiers reduced the scratch resistance of the coatings. Garlic (250 g) and nettle (200 g) significantly reduced scratch resistance, which may be related to the presence of plant particles with lower mechanical strength. Turmeric (300 g) exhibited slightly higher resistance but still noticeably lower than the reference sample. Privet (350 g) was the only sample close to the reference value, which may indicate its higher resistance to mechanical damage. However, considering the measurement error, it can be concluded that, the NET, GAR and TUR or GAR, TUR and PRI samples show similar levels of scratch resistance. Similarly, the scratch resistance of TUR and PRI samples are at a similar level to the reference sample.

#### 3.2.4. Cross-Cut Test

**Figure 12:**
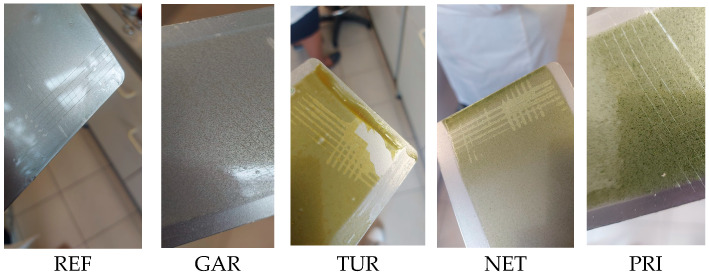
Cross-cut test patterns of epoxy coatings: unmodified epoxy, coating with garlic, coating with turmeric, coating with nettle, and coating with privet.

The same samples were further evaluated using the cross-cut test to assess adhesion and resistance to mechanical damage after modification. Representative images showing the incision patterns of the unmodified coating and those containing garlic, turmeric, nettle, and privet are presented in Figure 12.

The images were evaluated according to the cross-cut test classification system described above, in accordance with ISO 2409 [24]. Each sample was assigned a class according to the extent of coating detachment within the incised grid, ranging from Class 0 (no detachment) to Class 5 (severe detachment). The classification provides a standardized assessment of coating adhesion and mechanical durability. The detailed results of the cross-cut adhesion test for all samples are summarized in Table 7 below.

The cross-cut test results demonstrated that the unmodified epoxy resin and the coatings modified with garlic and privet exhibited excellent adhesion, classified as Class 0, indicating no coating detachment. In contrast, the coatings containing turmeric and nettle exhibited the lowest adhesion performance (Class 3), indicating partial coating detachment along the incision edges.

#### 3.2.5. Kӧnig Pendulum Hardness

**Figure 13:**
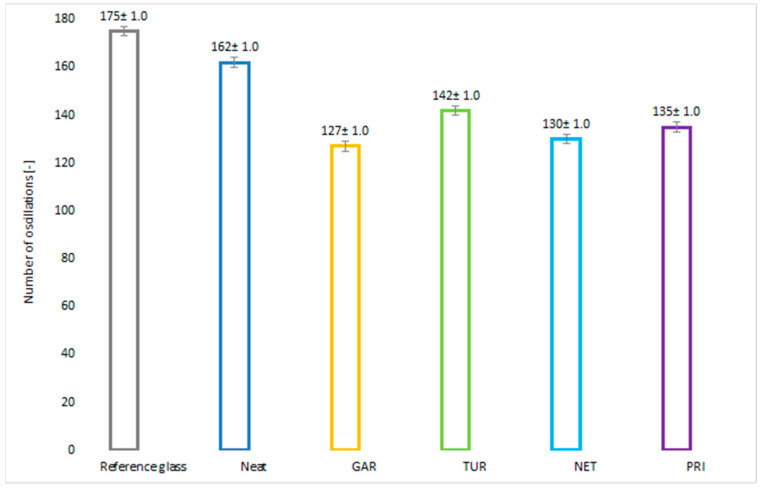
Number of Kӧnig pendulum oscillations on epoxy coatings with and without plant-based modifiers.

**Figure 14:**
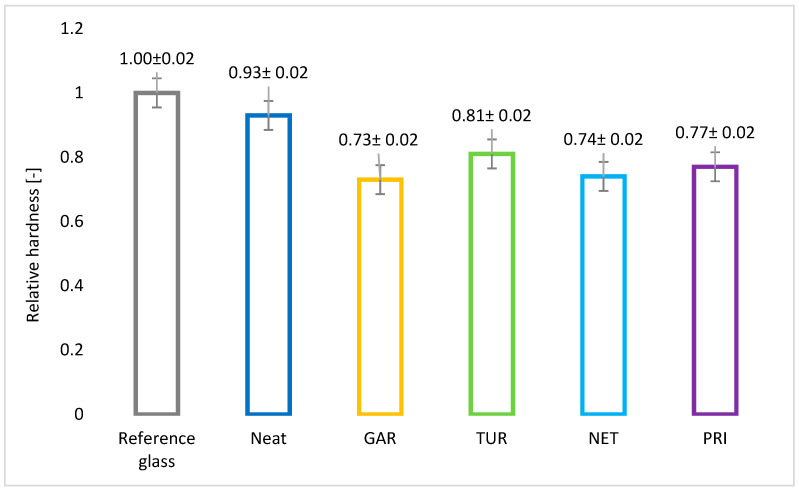
Kӧnig pendulum hardness of epoxy coatings with and without plant-based modifiers.

The test results are presented in Figure 13 and Figure 14. The reference glass (175 oscillations) served as the standard, representing the maximum attainable hardness in the test.

The neat epoxy resin (162 oscillations) reached a value close to that of the glass standard, confirming its good mechanical strength and the effectiveness of the crosslinking process. All modified samples exhibited lower hardness compared to the unmodified resin, suggesting that plant-based modifiers affect the flexibility of the coating. Among the modified coatings, turmeric exhibited the highest hardness (142 oscillations), indicating a moderate impact on this property, while garlic showed the lowest hardness (127 oscillations). Nevertheless, all tested coatings exhibited relatively high hardness values. Excessive hardness may lead to coating cracking under mechanical loading. In accordance with Qualicoat requirements, the number of oscillations should exceed 80 [40]. Taking into account the relative hardness values and their measurement error, it can be concluded that they are at a similar level for the modified samples, lower than for the reference sample. The reduction in hardness observed for the plant fillers-modified coating provides beneficial flexibility for applications ex-posed to mechanical stress.

#### 3.2.6. Gloss

**Figure 15:**
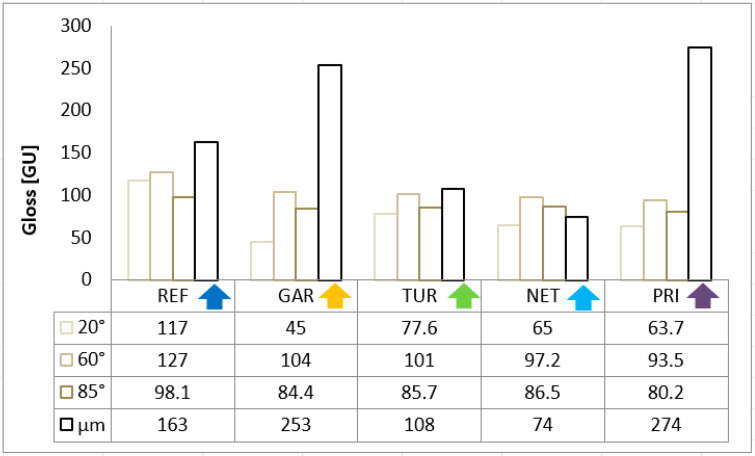
The gloss values of epoxy coatings measured at 20°, 60°, and 85°, together with the average coating thickness.

Gloss measurements obtained at 20°, 60°, and 85° enabled a comprehensive evaluation of surface reflectivity among different formulations. The results are presented in Figure 15.

The gloss values of all tested coatings measured at 60° exceed 90 GU, classifying them as high-gloss coatings. The reference sample exhibited the highest gloss, confirming its smooth surface. The addition of plant-based modifiers reduced gloss, especially at 20°, likely due to increased micro-roughness and light scattering. The lowest gloss values were observed for the garlic- and privet-modified coatings. Turmeric achieved the highest gloss among the modified samples, indicating good compatibility with the resin. The thickness of the tested coatings ranged from 74 to 274 µm, which is typical for solvent-free liquid coatings.

#### 3.2.7. Color

Colorimetric analysis was performed to assess the impact of plant-based additives on the visual properties of epoxy coatings. The evaluation was based on the CIELAB color space parameters (L, a, b*) and the total color difference (ΔE) relative to the reference sample. The results provide insight into both the intensity and the nature of the color shifts caused by the modifiers. The CIELAB color parameters and total color differences (ΔE) between the reference sample (REF) and the modified samples are presented in Table 8, Table 9, Table 10 and Table 11.

The colorimetric analysis shows that the addition of garlic (GAR) has a negligible effect on the optical properties of the coating. The total color difference (ΔE = 0.05) remains well below the threshold of visual perception, indicating that the sample retains the transparency and neutral tone of the reference. Minimal shifts in the L, a, and b* values confirm the absence of noticeable color change, making garlic an ideal additive for applications where color integrity is essential.

The addition of turmeric (TUR) caused a noticeable change in the color of the coating, as indicated by the total color difference (ΔE = 9.26). The significant increase in the b value suggests a strong yellow hue, while the decrease in L indicates a darker tone relative to the reference. These results confirm that turmeric acts as a vivid natural dye, visibly altering the appearance of the coating.

The addition of Urtica dioica (NET) moderately altered the color of the coating, with a total color difference (ΔE = 6.85) that is visually perceptible. The decrease in L indicates a slight darkening of the sample, while the increase in b and the negative a* value suggest a shift toward a greenish-yellow hue.

The addition of Ligustrum vulgare (PRI) caused the most significant color change among all tested plant-based materials, with a total color difference (ΔE = 15.04) that is clearly visible to the naked eye. The decrease in L indicates a darker surface, while the strongly negative a value reflects a shift toward green, and the high b* value indicates a pronounced yellow hue.

#### 3.2.8. Contact Angle Measurements

The measurement involved analyzing the angle formed between a water droplet and the sample surface, enabling the evaluation of its hydrophilic or hydrophobic character [41]. In general, surfaces can be classified according to their contact angle as follows: angles below 90° indicate a hydrophilic surface (easily wetted by water), angles above 90° correspond to a hydrophobic surface (poorly wetted by water), angles close to 0° represent very strong water adhesion (e.g., glass), and angles approaching 180° indicate extreme hydrophobicity (e.g., superhydrophobic surfaces).

**Figure 16:**
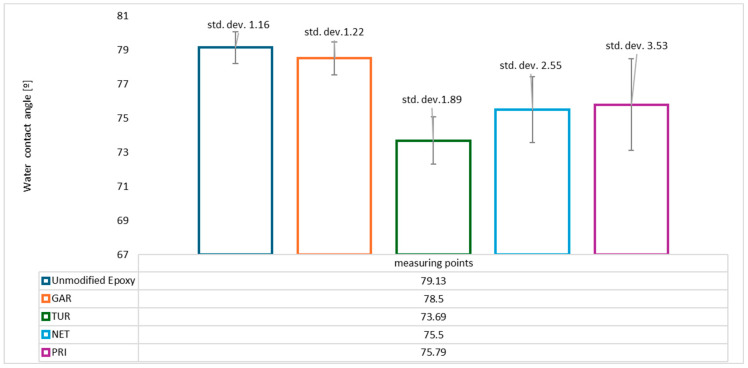
The water contact angle values for the tested coatings.

The results of the measurements are summarized in Figure 16.

The results indicate that all tested coatings exhibit hydrophilic behavior, as their mean contact angles are below 90°. The unmodified coating shows the highest average contact angle (79.13°). The modified coatings—garlic (78.50°), turmeric (73.69°), privet (75.79°), and nettle (75.50°)—exhibit slightly lower contact angles, indicating improved wettability. However, considering the standard deviations, it can be concluded that, with the exception of turmeric, the remaining values are at a similar level to the reference sample. In the case of hydrophilic surfaces, the contact angle may decrease [42] or increase [43]. Some sources indicate that differences become noticeable at roughness values above Ra = 16 µm [44]. In our case, the difference in roughness is less than 5 µm, so it should not have a significant effect on the water contact angle values.

#### 3.2.9. Assessment of Antimicrobial Activity

**Figure 17:**
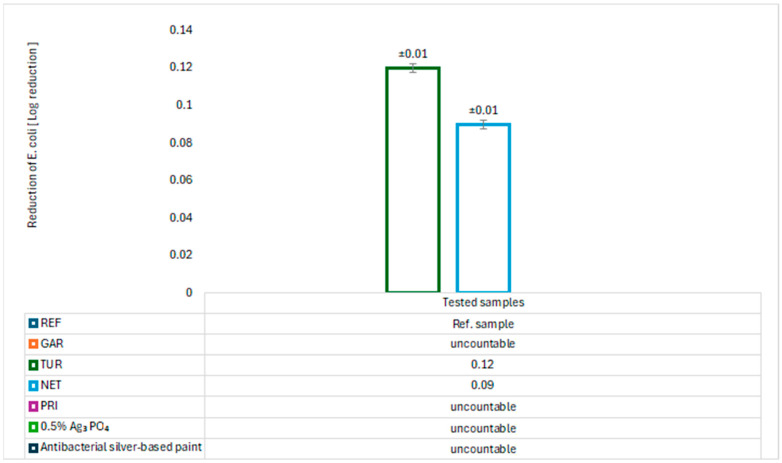
Logarithm reduction in *E. coli* bacteria.

**Figure 18:**
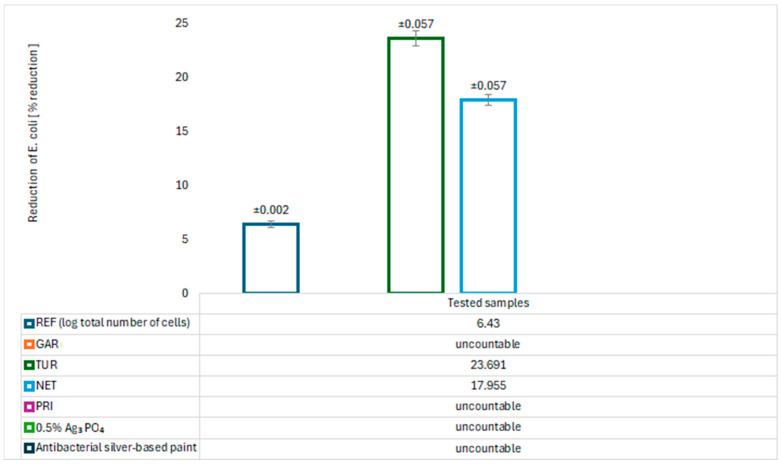
Percent reduction in *E. coli* bacteria.

**Figure 19:**
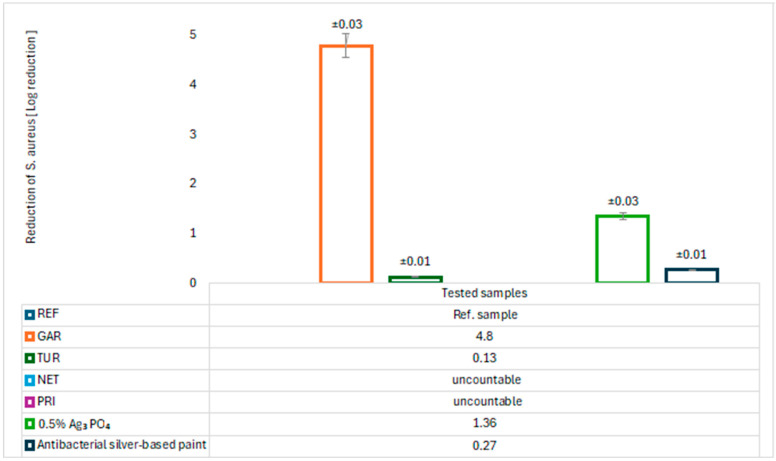
Logarithm reduction in *S. aureus* bacteria.

**Figure 20:**
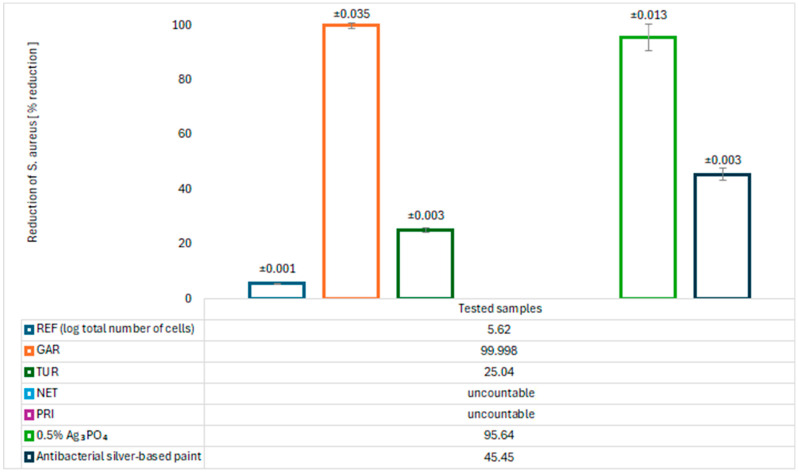
Percent reduction in *S. aureus* bacteria.

The antimicrobial properties of the modified coatings were evaluated by assessing bacterial growth on agar plates. The tests were conducted using two model bacterial strains: *Escherichia coli* (Gram-negative) and *Staphylococcus aureus* (Gram-positive). The results are presented in Figure 17, Figure 18, Figure 19 and Figure 20.

After 24 h of incubation, abundant growth of *E. coli* was observed on the GAR and PRI samples, as well as on the commercial paint, indicating no bacteriostatic effect. In contrast, the TUR and NET samples demonstrated a significant ability to reduce the number of *E. coli* colonies, with values of 23.691 ± 0.057 (TUR) and 17.955 ± 0.057 (NET), respectively. These results confirm their bacteriostatic activity against *E. coli*. For *S. aureus*, neither the NET nor the PRI coatings showed reduction activity. The TUR sample, however, demonstrated a measurable bacteriostatic effect, while the commercial paint exhibited a slightly stronger inhibitory effect on *S. aureus*. The antibacterial effect of turmeric can be attributed to the presence of curcumin, which has been reported to inhibit bacterial proliferation [11]. The antibacterial properties of nettle are associated with bioactive compounds such as flavonoids, tannins, caffeic acid, and catechins, which inhibit the growth and reproduction of pathogenic bacteria [12,13].

An almost complete absence of *Staphylococcus aureus* colony growth was observed on the GAR coating surface. The quantitative assessment of antibacterial activity revealed that the coating containing 5% garlic modifier exhibited exceptionally high effectiveness against *S. aureus*. The calculated log_10_ reduction of 4.80, corresponding to a bacterial reduction of 99.998%, confirms the strong antimicrobial performance of the modified coating. These results indicate the bactericidal activity of garlic against Gram-positive bacteria. A relatively low concentration of garlic can significantly reduce microbial contamination, making this bio-based modification a promising alternative to synthetic biocides and precious metals such as silver in antimicrobial surface applications. For comparison, Figure 17, Figure 18, Figure 19 and Figure 20 also presents the results of tests on the antibacterial activity of silver-based epoxy coatings, which exhibited a weaker antibacterial effect against *S. aureus* compared to garlic. The coating containing 0.5% Ag_3_PO_4_ reduced 95.640% of *S. aureus* bacteria, while the commercial coating reduced it by only 45.450%. The strong antibacterial activity of garlic is attributed to the presence of allicin, which can interact with thiol (–SH) groups in microbial proteins, leading to their damage and disruption of their functions [14].

#### 3.2.10. Scanning Kelvin Probe Measurements

To elucidate the mechanism underlying the antibacterial activity of the coatings, measurements were performed using the Scanning Kelvin Probe (SKP) technique, which enabled the determination of the surface potential distribution and its relationship to microbial colonization [15].

**Figure 21:**
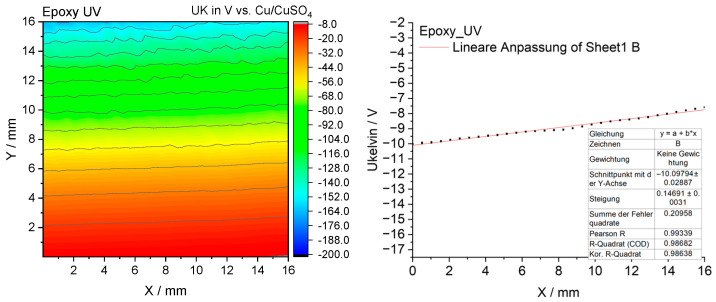
The surface potential map obtained by SKP for the unmodified epoxy coating (reference sample).

**Figure 22:**
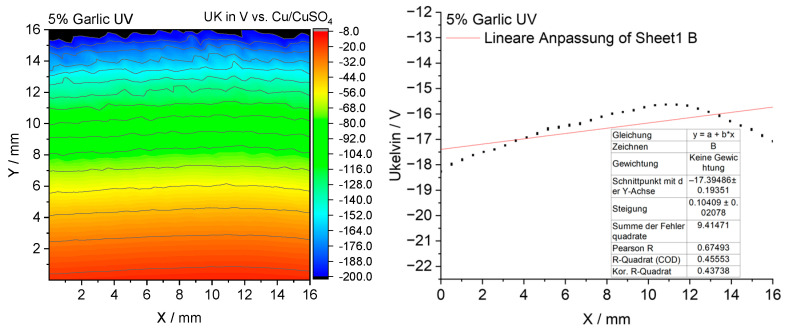
The surface potential map obtained by SKP for the epoxy coating containing 5% garlic modifier.

**Figure 23:**
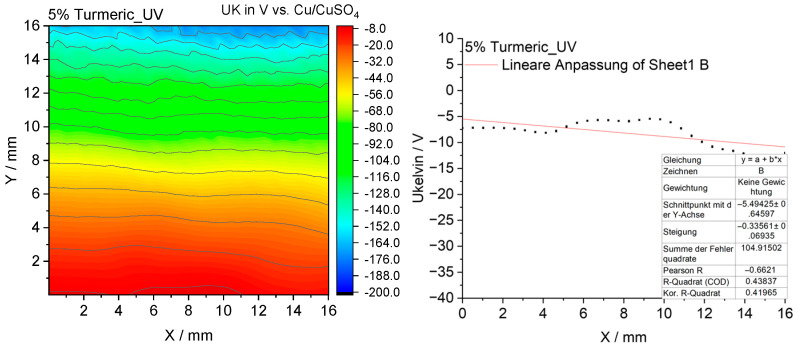
The surface potential map obtained by SKP for the epoxy coating containing 5% turmeric modifier.

**Figure 24:**
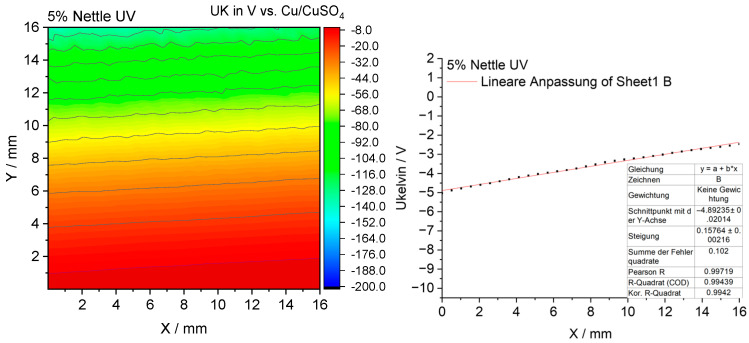
The surface potential map obtained by SKP for the epoxy coating containing 5% nettle modifier.

The SKP surface potential maps of the investigated coatings are presented in Figure 21, Figure 22, Figure 23 and Figure 24.

The epoxy reference coating shows a variation in surface potential, ranging from approximately −7.6 mV (red) to −166 mV (dark blue), suggesting a potential gradient along the *Y*-axis (Figure 21). The equipotential lines are uniform and show no abrupt changes, indicating a relatively homogeneous surface exhibiting a distinct potential gradient. This coating showed neither significant electrochemical protection nor notable antimicrobial activity.

**Figure 25:**
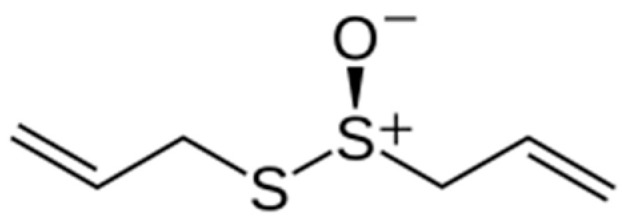
The chemical structure of allicin.

The garlic-modified coating exhibited pronounced antimicrobial activity against *S. aureus*. The corresponding SKP map revealed more negative potentials compared to the reference sample, indicating a surface modification induced by the garlic additive (Figure 22). This suggests that the antimicrobial effect may arise mainly from chemical inactivation of microorganisms rather than from electrochemical barrier properties. This interpretation supports the hypothesis that garlic acts primarily through bioactive sulfur-containing compounds such as allicin (Figure 25), which contain ionic groups capable of interacting strongly with bacterial cell membranes [15].

The turmeric-modified coating also shows a more negative and more homogeneous potential profile than the reference sample (Figure 23). Combined with the known properties of curcumin, this suggests a dual antimicrobial mechanism involving both barrier-based and chemical activity. The nettle-modified coating also displays a slightly more negative potential, indicating a moderate barrier effect (Figure 24). However, its antimicrobial efficacy in microbiological tests was less pronounced compared to the turmeric- and garlic-modified samples, due to its action mainly as an antioxidant with relatively weak antimicrobial performance. Overall, the SKP results reveal that increasingly negative surface potentials correlate with enhanced antibacterial performance, except for the antioxidant-dominated nettle system.

## 4. Conclusions

This study demonstrated that incorporating plant-based additives into UV-curable epoxy coatings significantly influences their antimicrobial, mechanical, and surface properties. Among the tested modifiers, garlic proved to be the most effective against Staphylococcus aureus, achieving nearly complete bacterial reduction (~4.8 log; ≈99.998%), while maintaining proper curing of the epoxy matrix as confirmed by FT-IR analysis. The antibacterial action of the coatings was species-selective, with turmeric and nettle showing higher activity against E. coli, whereas garlic and privet were less effective in this regard. Despite the enhanced antimicrobial functionality, the inclusion of natural fillers generally reduced coating hardness and scratch resistance, indicating a trade-off between mechanical durability and biological efficacy. Garlic and privet maintained excellent adhesion to the substrate, while turmeric and nettle slightly decreased coating cohesion. All plant additives increased surface roughness and reduced gloss, most notably for garlic and privet, which correlated with a more heterogeneous topography. The coatings remained hydrophilic, and the observed variations in wetting behavior were moderate. Color stability was preserved in garlic-modified coatings, whereas turmeric and privet imparted a visible yellow or green hue, respectively. Surface potential mapping indicated more negative potentials for samples with higher antibacterial activity, suggesting a mainly chemical interaction mechanism between garlic compounds and bacterial membranes, although this interpretation should be treated with caution. Garlic-modified epoxy coatings offer a promising bio-based alternative to conventional silver-containing systems for reducing S. aureus contamination on polymer surfaces, with the advantage of eliminating synthetic biocides. However, the results are limited to one resin type, a single additive concentration (5 wt.%), and short-term ISO 22196 testing. Further research should focus on the durability of the antibacterial effect, optimization of filler dispersion, dose–response studies, and compatibility with different resin systems and photoinitiators. Such investigations could confirm the potential of bio-modified epoxy coatings as safe, functional, and environmentally sustainable materials for use in public, medical, and consumer applications.

## Figures and Tables

**Table 1:** The qualitative/quantitative composition of the coatings.

Component/ Symbol of Coating	Epoxy Resin, wt.%	Photoinitiator wt.%	GAR wt.%	TUR wt.%	NET wt.%	PRI wt.%
REF (EP)	98.0	2.0	-	-	-	-
GAR	93.0	2.0	5.0	-	-	-
TUR	93.0	2.0	-	5.0	-	-
NET	93.0	2.0	-	-	5.0	-
PRI	93.0	2.0	-		-	5.0

**Table 2:** The CIELAB color parameters and total color difference (ΔE) before and after the weathering test of the garlic (GAR) sample.

GAR			
Parameter	Before Weathering	After Weathering	Δ (Before − After)
L*	72.13	73.43	1.30
a*	7.82	8.11	0.29
b*	24.77	23.20	1.57
			ΔE* = 2.06

**Table 3:** The CIELAB color parameters and total color difference (ΔE) before and after the weathering test of the turmeric (TUR) sample.

TUR			
Parameter	Before Weathering	After Weathering	Δ (Before − After)
L*	52.70	57.37	0.33
a*	24.78	21.28	3.50
b*	51.96	42.70	9.26
			ΔE* = 9.90

**Table 4:** The CIELAB color parameters and total color difference (ΔE) before and after the weathering test of the nettle (NET) sample.

NET			
Parameter	Before Weathering	After Weathering	Δ (Before − After)
L*	42.10	48.37	6.27
a*	−3.31	−1.42	1.89
b*	18.48	16.88	1.60
			ΔE* = 6.74

**Table 5:** The CIELAB color parameters and total color difference (ΔE) before and after the weathering test of the privet (PRI) sample.

PRI			
Parameter	Before Weathering	After Weathering	Δ (Before − After)
L*	50.06	54.60	4.54
a*	−4.02	−2.24	1.78
b*	22.73	15.31	7.42
			ΔE* = 8.88

**Table 6:** TGA results of used plant fillers.

Filler Symbol	Decomposition Temperature Ranges [°C]/wt.% Loss	DTG Max [°C]	Total wt.% Loss at 600 °C
GAR	25–150/6	100	71.8
	150–270/36	225	
	270–600/29.8	315	
TUR	25–190/7	90	70.9
	190–600/63.9	290	
NET	25–165/7	70	64.2
	165–600/57.2	310	
PRI	25–140/6	80	75.2
	140–600/69.2	310	

**Table 7:** Summary of cross-cut adhesion test results for epoxy coatings modified with different plant-based additives.

Sample	ISO Class [24]	Evaluation Description
Neat	0	No coating damage
GAR	0	No coating damage
TUR	3	Squares partly or fully damaged, 15–35% coating detachment
NET	3	Squares partly or fully damaged, 15–35% coating detachment
PRI	0	No coating damage

**Table 8:** The CIELAB color parameters and total color difference (ΔE) between the reference sample (REF) and the garlic-modified sample (GAR).

GAR			
Parameter	Standard	Sample Garlic	Δ (Sample − Standard)
L*	39.67	46.52	0.02
a*	0.06	0.08	−0.02
b*	0.56	2.04	0.04
			ΔE* = 0.05

**Table 9:** The CIELAB color parameters and total color difference (ΔE) between the reference sample (REF) and the turmeric-modified sample (TUR).

TUR			
Parameter	Standard	Sample Curcuma	Δ (Sample − Standard)
L*	39.67	33.56	6.27
a*	0.06	0.37	−0.31
b*	0.56	7.37	−6.81
			ΔE* = 9.26

**Table 10:** The CIELAB color parameters and total color difference (ΔE) between the reference sample (REF) and the Urtica dioica-modified sample (NET).

NET			
Parameter	Standard	Sample Urica Dioica	Δ (Sample − Standard)
L*	39.67	38.18	1.31
a*	0.06	−2.15	2.2
b*	0.56	6.91	−6.35
			ΔE* = 6.85

**Table 11:** The CIELAB color parameters and total color difference (ΔE) between the reference sample (REF) and the Ligustrum vulgare-modified sample (PRI).

PRI			
Parameter	Standard	Sample Ligustrum	Δ (Sample − Standard)
L*	39.67	36.78	2.89
a*	0.06	−3.76	3.82
b*	0.56	14.82	−14.26
			ΔE* = 15.04

## Data Availability

The original contributions presented in this study are included in this article. Further inquiries can be directed to the corresponding authors.
